# A Bayesian model for control strategy selection against *Plasmopara viticola* infections

**DOI:** 10.3389/fpls.2023.1117498

**Published:** 2023-07-20

**Authors:** Lorenzo Valleggi, Giuseppe Carella, Rita Perria, Laura Mugnai, Federico Mattia Stefanini

**Affiliations:** ^1^ Department of Statistics, Computer Science, Application (DISIA), University of Florence, Florence, Italy; ^2^ Department of Agronomy, Food, Environmental and Forestry (DAGRI), University of Florence, Florence, Italy; ^3^ Council for Agricultural Research and Economics, Research Centre for Viticulture and Enology, Arezzo, Italy; ^4^ Department of Environmental Science and Policy, University of Milan, Milan, Italy

**Keywords:** disease modeling, pesticides reduction, downy mildew, Bayesian modeling, utility function, sustainability

## Abstract

Plant pathogens pose a persistent threat to grape production, causing significant economic losses if disease management strategies are not carefully planned and implemented. Simulation models are one approach to address this challenge because they provide short-term and field-scale disease prediction by incorporating the biological mechanisms of the disease process and the different phenological stages of the vines. In this study, we developed a Bayesian model to predict the probability of *Plasmopara viticola* infection in grapevines, considering various disease management approaches. To aid decision-making, we introduced a multi-attribute utility function that incorporated a sustainability index for each strategy. The data used in this study were derived from trials conducted during the production years 2018-2020, involving the application of five disease management strategies: conventional Integrated Pest Management (IPM), conventional organic, IPM with substantial fungicide reduction combined with host-defense inducing biostimulants, organic management with biostimulants, and the use of biostimulants only. Two scenarios were considered, one with medium pathogen pressure (Average) and another with high pathogen pressure (Severe). The results indicated that when sustainability indexes were not considered, the conventional IPM strategy provided the most effective disease management in the Average scenario. However, when sustainability indexes were included, the utility values of conventional strategies approached those of reduced fungicide strategies due to their lower environmental impact. In the Severe scenario, the application of biostimulants alone emerged as the most effective strategy. These results suggest that in situations of high disease pressure, the use of conventional strategies effectively combats the disease but at the expense of a greater environmental impact. In contrast to mechanistic-deterministic approaches recently published in the literature, the proposed Bayesian model takes into account the main sources of heterogeneity through the two group-level effects, providing accurate predictions, although precise estimates of random effects may require larger samples than usual. Moreover, the proposed Bayesian model assists the agronomist in selecting the most effective crop protection strategy while accounting for induced environmental side effects through customizable utility functions.

## 1 Introduction

*Plasmopara viticola* is a heterothallic oomycete that is the causal agent of downy mildew (DM), one of the most severe diseases of grapevines in many viticultural areas of the world (Wong et al., 2001). Its life cycle starts in autumn when oospores enter their overwintering stage in infected leaves on the ground. At the beginning of spring, zoospores, released by macrosporangia produced by oospore germination, are distributed by rain and wind on new leaves, shoots and later, clusters of the vine. New zoospores are produced by asexual reproduction, and this occurs throughout the growing season infecting new tissues, often leading to heavy economic losses (Gessler et al., 2011). To prevent DM infections, fungicide applications are usually required, many applications of fungicides are usually necessary to prevent DM infections, but some of those applied in agriculture can have a significant impact on the environment (Shunthirasingham et al., 2010) and human health (Kab et al., 2017). There is a heavy impact of fungicide strategies also in organic viticulture (ORG), where mainly copper-based products are applied (Dagostin et al., 2011), as copper can accumulate in the soil and damage the microflora and microfauna (Cavani et al., 2016). That is why, based on Regulation (EU) 2018/1981 of 13 December 2018, the use of copper is strictly limited. The EU is making many efforts to reduce the impact of fungicides on the environment. One of the strategies proposed in the literature enhances the resilience capacity of the grapevine to reduce the use of fungicides with a potential environmental impact.

Perria et al. (2022) promoted the use of “GreenGrapes” strategies, including integration or substitution of products based on plant, seaweed or yeast extracts that guarantee greater environmental sustainability in viticulture, with a good or acceptable protection level compared to conventional pesticides, both in ORG and IPM management. The latter context sees the integration of defense induction activity alongside the more frequently used direct antifungal activity, and the application of an efficient Vite.net system (a Decision Support System [DSS] developed by Horta s.r.l., providing daily information updates to aid careful scheduling of antifungal treatments). This system predicts the probability of infection events, leading to optimal scheduling of the strategies, enabling a move toward more environmental sustainability in viticulture.

Many simulation models to provide short-term and field-scale DM predictions have been developed in recent years, one of the most recent ones being proposed by Bove et al. (2020a). These authors developed the model considering all of the biological mechanisms of the disease process and the different phenological stages of the vines. They simulated the infection that occurred on healthy foliage, which generated a sporulation site producing the secondary infections. Also, the infection on clusters is simulated as a rate, the function of a specific transmission coefficient. This model reproduces the disease kinetic (number of diseased sites) based on tuning parameters, but as the authors declare, many simplifications were made, especially on cluster infections, due to the lack of information in the literature and the inherent complexity. Also, they considered a steady-state system, where plant structure and microclimatic conditions were stable. Brischetto et al. (2021) extended this simulation model using findings from previous studies [(Caffi et al., 2016), (Magarey et al., 2005), (Bove et al., 2020b), (Lalancette, 1988)] to develop a proper DSS, so that scouting of the vineyard and monitoring the environmental conditions could give information about the expected sporangia development, sporangia availability, and the relative severity of lesions, and thus determine secondary infection cycles. Other authors (Chen et al., 2020) compared statistical models and machine learning algorithms to predict infection by DM in terms of incidence and severity, using field scouting and climate variables as inputs. The results were used by the authors to evaluate the potential reduction in the number of fungicide applications.

In this work, we present a novel approach to address the challenge of predicting *Plasmopara viticola* incidence under different agronomic treatment strategies using Bayesian models and utility functions. This research aims to bridge a significant gap in the current literature, because the use of Bayesian models and utility functions is still not widespread in the agronomic field, especially in *Plasmopara viticola* studies. Our proposal assimilates expert knowledge at three levels: the first deals with the structure of the statistical model, and the second with the elicitation of prior distributions for model parameters. In the third level, the development of utility functions makes it possible to consider the preferences and priorities of decision-makers in a quantitative way, while evaluating treatment strategies. This novel aspect of our research empowers stakeholders to make more informed decisions about strategies by incorporating their subjective preferences, treatment efficiency and environmental implications at the same time.

## 2 Materials and methods

### 2.1 Experiment description

All the details about the original experiment, such as vine age, vine spacing, pruning and training system, and also on products used, spraying schedule and dates, spraying equipment and volume per ha are reported in the paper by Perria et al. (2022), which aimed to evaluate five disease management strategies. These were: Integrated Pest Management (IPM) (“Strategy 1”), the IPM management modified by a reduction in fungicides and use of plant defense supporting biostimulants (IPM-GG) (“Strategy 2”), organic management (ORG) (“Strategy 3”), organic management with reduced copper application, and plant defense supporting biostimulants (ORG-GG) (“Strategy 4”) and only biostimulants application (“Strategy 5”). Strategy 5 was considered in this analysis as the experimental control because it did not include fungicides. These crop protection strategies were applied over three years from 2018 to 2020, in a cultivar Sangiovese vineyard located in the Chianti Classico wine district (Perria et al., 2022). The trial was applied to an area of 50,000 *m* ^2^ which was divided into 5 blocks each 10,000 *m* ^2^. These blocks were environmentally and pedologically homogeneous. For each strategy, four sub-plots with the size of eight vines were randomly selected at the beginning of the experiment, for a survey of disease symptoms on leaf and bunch.

The survey was conducted in each sub-plot, where 100 leaves and 100 bunches (if sufficient numbers were present) were sampled at different dates to measure the disease incidence and severity (EPPO guidelines). The survey was conducted from May to the end of July each year, but the analysis carried out in the present study considered only disease parameters obtained at the last time point in each year at the phenological phase BBCH 85-89.

### 2.2 Model specification

A Bernoulli random variable describes the presence, Y=1 , or absence, Y=0 , of disease, i.e. if the observational unit i∈{1,2,…,n} is infected under strategy k∈{0,1,2,…,K−1} at the end of year t∈{1,2,…,T} , thus $Y_{i}∼Bern\left(\pi_{i}\right)$ . Following Gelman and Hill notation (2007, chap. 14), a logistic regression model has been defined as:


(1) $$Pr\left(Y_{i}=1\right)=logit^{-1}\left(\alpha_{t\left[i\right]}+\gamma_{k\left[i\right],t\left[i\right]}+\beta_{k\left[i\right]}\right)$$

where betas are (fixed) effects due to the strategy applied and their initial distribution is defined by marginally independent uniform distributions $\beta_{k}\propto U_{\left(-\infty ,+\infty \right)}$ (see Gelman et al., 2006); the notation $\beta_{k\left[i\right]}$ refers to an element in the vector of betas whose index k[i] depends on statistical unit *i*; the random fluctuation due to year *t* is described by $\alpha_{t\left[i\right]}$ ; alphas are normal and marginally independent in the initial distribution with $\alpha_{t}∼N\left(0,\sigma_{\alpha }\right)\text{ and }\sigma_{\alpha }∼Half-t\left(3,0,2.5\right)$ , which is (half of) a Student-t distribution defined on positive reals, with 3 degree of freedom, location 0 and scale 2.5; gammas are random variables describing year-specific fluctuations of strategies around the average represented by betas (Gelman and Hill, 2007), and the initial distribution is defined by marginally independent components $\gamma_{k,t}∼N\left(0,\sigma_{\gamma_{k}}\right)\text{ with }\sigma_{\gamma_{k}}∼Half-t\left(3,0,2.5\right),k=0,\ldots ,4$ . The prior distributions for the standard deviation and its hyperparameters were weakly informative, so that data dominate on expert prior belief in the posterior distribution.

The above model features were discussed with the experts and it was recognized how the disease may start with different pressures every year due to the dependence on environmental conditions. Furthermore, leaves also change every year, thus important features of the statistical unit, the plant, represent a source of variability in the response to be taken into account. Similarly, each strategy may have slightly different effects across years, as described by the considered parameters gammas. The baseline (model intercept) is $\beta_{0}$ , i.e. *Strategy 5* in the original study, whose components are only plant-defense-supporting biostimulants. The outputs of the model are the Odds ratios (ORs), which are the result of exponentiating the parameters in a logistic regression model. The latter represents the log odds of an event occurring (in this case, the disease event) compared to the probability of the event not occurring in a specific category (e.g., Strategies). ORs are actually the probability of an event occurring between two different categories (Strategy 1 vs Strategy 2). If the value is greater than 1, it means that the probability of the event is higher in the category at the numerator of the ratio, while if the value is less than 1, it indicates that the probability of the event is higher in the category at the denominator of the ratio.

The final distribution (also called *a-posteriori* distribution) of model parameters after learning from field data has been approximated by Markov Chain Monte Carlo simulation (Van de Schoot et al., 2021), see results. Four strategies (Strategies 1-4) were compared to the reference strategy (Strategy 5) and expected values and credible intervals of these effects were calculated. Nevertheless, side effects specific to each strategy may reduce/increase the appeal of strategies, for example, because of the magnitude of secondary effects induced in the soil. For this reason, a utility function U() has been defined in order to support the choice of strategy in future fieldwork.

### 2.3 Utility function

A utility function U() was defined to find the optimal phytosanitary strategy for crop protection in a hypothetical next year by joint evaluation of the probability of infection for one leaf and the sustainability of the selected strategy. A number of attributes were selected to describe the future consequences of a selected strategy and the uncertainty on the value taken by the attributes in the following year was described by predictive and prior-predictive distributions. The Simple Multi-Attribute Rating Technique (SMART) (Edwards, 1977) is the multi-attribute framework adopted here to define a utility function U() that compares alternative crop protection strategies by rating attributes $a_{1},\ldots ,a_{j},\ldots ,a_{m}$ on a natural scale. The value of each sub-utility function $u_{j}\left(a_{j,k}\right)$ dealing with attribute $a_{j}$ under crop protection strategy *k* was multiplied by weight *w_j_*, where weights are subject to $\sum_{j}w_{j}=1$ . The importance of an attribute $a_{j}$ is reflected in a high value of its weight *w_j_*. The utility value U(k) of the crop protection strategy *k* is calculated by a linear additive model of all sub-utility functions and normalized to range from 0 to 1:


$$U\left(k\right)=\sum_{j=1}^{m}w_{j}\;u_{j}\left(a_{j,k}\right),k=1,2\ldots K$$

where 0 is the worst and 1 is the best value of U(k) .

The first attribute ( $a_{1}$ ) is the probability of infection of one leaf in the next year. Subsequent attributes ( j=2,…,8 ) describe the sustainability in terms of environmental impact and toxicological effects, in particular they are:

- *ɑ*_2_: the Human Tox score that defines the impact of toxic substances on human health;
- *ɑ*_3_: the Treatment Frequency Index, determined by the absolute frequency of fungicide applications;
- *ɑ*_4_: the Carbon Footprint, based on the amount of greenhouse gases produced;
- *ɑ*_5_: the Carbon sequestration index, which is the amount of carbon seized by plant tissues;
- *ɑ*_6_: the Ecological Footprint, which quantifies the biologically productive land and aquatic surface needed to provide resources and absorb emissions for the production of a certain good or service;
- *ɑ*_7_: the Eco Tox Score, to evaluate the eco-toxicological risk on the health of the aquatic and terrestrial ecosystems, due to synthetic chemicals used in the field;
- *ɑ*_8_: the Water Footprint, which is based on the water consumption of the production process;

Details on the above attributes are contained in Perria et al. (2022).

The sample space of each environmental index (listed above) was divided into four classes from 0 to 3, where the best class is labeled as 0 and the worst as 3. The sub-utility function *u*_1_ depends on ${\tilde{\phi }}_{k}$ , which is the probability of infection for one leaf next year under strategy *k*

as described by the Bayesian predictive distribution conditioned to observed data. The sub-utility function *u*_1_ has been elicited as a negative exponential function:


$$u_{1}\left(k\right)={\left(1-{\tilde{\phi }}_{k}\right)}^{\delta }\;I_{\left[0,0.1\right]}(\tilde{\phi })$$

where δ is a positive tuning parameter chosen by the expert. The value of δ modifies the rate at which the utility decreases with increasing probability of infection ( $\tilde{\phi }$ ); if δ>1 it implies a faster decrease while if δ<1 it implies a slower decrease in utility value. More conservative experts tend to set δ values greater than 1 to prioritize strategies with high protection. In this case, we set δ =0.4, which was deemed a suitable value for this analysis. A threshold of 0.1 was established such that when the percentage of infected leaves exceeds 10% (based on a sample of 100 leaves), the utility of the attribute representing the probability of infection is set to zero. This threshold was determined based on input from our expertise, who believed that strategies under consideration would not enable recovery of the vineyard if the infected leaf percentage exceeded this threshold. This threshold is subjective and can be adjusted by agronomists depending on the disease’s potential for spread and on personal evaluation of risk. In order to achieve this threshold, indicating function was defined ( $I_{\left[0,0.1\right]}$ ), which becomes zero when the probability of infection exceeds the threshold, and otherwise becomes 1. The elicitation of additional sub-utility functions was not performed in the same manner as the primary utility function, as a simple rescaling of their values to a range from 0 to 1 was judged flexible enough by the expert:


$$u_{j}\left(k\right)=\frac{a_{j}^{*}-a_{j,k}}{a_{j}^{*}-a_{j}^{0}}\text{ with }j=2,3,\ldots ,8$$

where $a_{j}^{*}$ is the maximum and $a_{j}^{0}$ the minimum for attribute $a_{j}$ on the original scale.

The weights $w_{j},\;j=1,2,\ldots ,8$ were defined as follows: $w_{1}=8/14$ and $w_{j}=6/98$ for each attribute after the first.

In order to rank the five considered strategies in terms of utility, expected values E[U(k)|D] were calculated for each strategy given the collected data D , and the best strategy in a given scenario was found as the value *k* determining the expected utility maximum:


(2) $$k^{*}=\arg\;\underset{k}{\max}E[U\left(k\right)|D]$$

where the expectation of U(k) is calculated with respect to the distribution of the attributes $a_{1,k},\ldots ,a_{8,k}$ describing the consequences of the adopted strategy in the future:


$$p({\tilde{\phi }}_{k}|D)\cdot \prod_{j=2}^{8}p({\tilde{a}}_{j}|\alpha_{j},k)$$

where $p({\tilde{a}}_{j}|\alpha_{j},k)$ is the elicited prior-predictive distribution of the future score ${\tilde{a}}_{j}$ for attribute *j* under strategy *k*: these are members of the Multinomial-Dirichlet family of distributions with parameter vector $\alpha_{j}$ (see below); $p({\tilde{\phi }}_{k}|D)$ is the predictive distribution for the future probability of infection of one leaf under strategy *k* given the experimental data. Equivalently, equation (2) may be expanded as follows:


$$k^{*}=\arg\;\underset{k}{\max}\left\{w_{1}\int {\left(1-{\tilde{\phi }}_{k}\right)}^{\delta }\;p({\tilde{\phi }}_{k}|D)\cdot d{\tilde{\phi }}_{k}+\sum_{j=2}^{m}w_{j}\int v_{j}\left({\tilde{a}}_{j,k}\right)\;p({\tilde{a}}_{j}|\alpha_{j},k)\;da_{j,k}\right\}$$

thus $k^{*}$ is the strategy that the decision-maker (in this case the agronomist) should apply in the following year of grapevine production Smith, 2010).

### 2.4 Computing and model diagnostic

Markov Chain Monte Carlo simulation was performed using *rstan and brms* packages (Bürkner, 2017b; Carpenter et al., 2017) in order to fit a Bayesian Linear Mixed Model (GLMM) using a No-U-Turn sampler, which is an adaptive version of Hamiltonian Monte Carlo sampling (HMC) (Bürkner, 2017a). The predictive probability of infection for each year and strategy was estimated using kernel density curves, which were used to perform a predictive check. The graphs depict the comparison between the predicted probability of infection by the model and the observed average. This comparison is performed to assess the compatibility of the predicted mean of the new observations with the observed one, and to examine the distribution of the new observations. The highest density interval (HDI) was also computed, which indicates the range of values that are most plausible for a given parameter based on the posterior distribution. In this case, the HDI represents the range with 80% of the posterior density. Model quality and fit were evaluated using trace plots, which were among the output diagnostic tools used. Continuous residuals were obtained by calculating residuals using the *DHARMa* R package which uses the inverse of the cumulative distribution function of the standard normal to evaluate the residuals in the generalized mixed linear model (Gelman et al., 1995; Dunn and Smyth, 1996). Traceplot is a graphical diagnostic tool applied to each parameter of the posterior sample generated in Bayesian statistical analysis, and is commonly used to check the validity and reliability of the posterior estimates generated by the MCMC algorithm. The main use of traceplot is to assess the convergence and mixing properties of the MCMC algorithm. If the MCMC algorithm has converged, the traceplot should show a stable pattern over time, with little variability in the posterior samples. Additionally, traceplot can also help to identify any potential issues with the MCMC 8 algorithm, such as poor mixing, which can affect the accuracy of the posterior estimates (Van de Schoot et al., 2021). A complete list of all packages used in this work is available in the **Supplementary Material**.

### 2.5 Scenario-building

The proposed Bayesian model can be exploited to predict the probability of infection at the end of the season each year for one randomly sampled leaf, given a selected strategy among those investigated. As a relevant amount of variability depends on features specific to each year, several scenarios may be defined. In particular, two main scenarios were selected: in the first one, average environmental fluctuations to represent an average disease pressure for DM development were considered, while in the second scenario, the best environmental fluctuations for DM development to represent a high pressure were selected, which corresponds to the worst situation for the farmer. Through the estimation of the group-level parameters, it was possible to predict infection probability under each strategy. Each scenario refers to a value of parameter α , where $\alpha ∼N\left(0,\sigma_{\alpha }\right)$ for average pressure, and in particular $\alpha_{hp}=+2\cdot \sigma_{\alpha }$ for high disease pressure. The average pressure scenario could have been associated to $\alpha_{ap}=0$ but, given the limited number of considered years, we preferred to set $\alpha_{ap}$ to the value estimated in the year 2020, which is the closest value to zero among the three available years.

## 3 Results

### 3.1 Descriptive statistics

**Figure 1:**
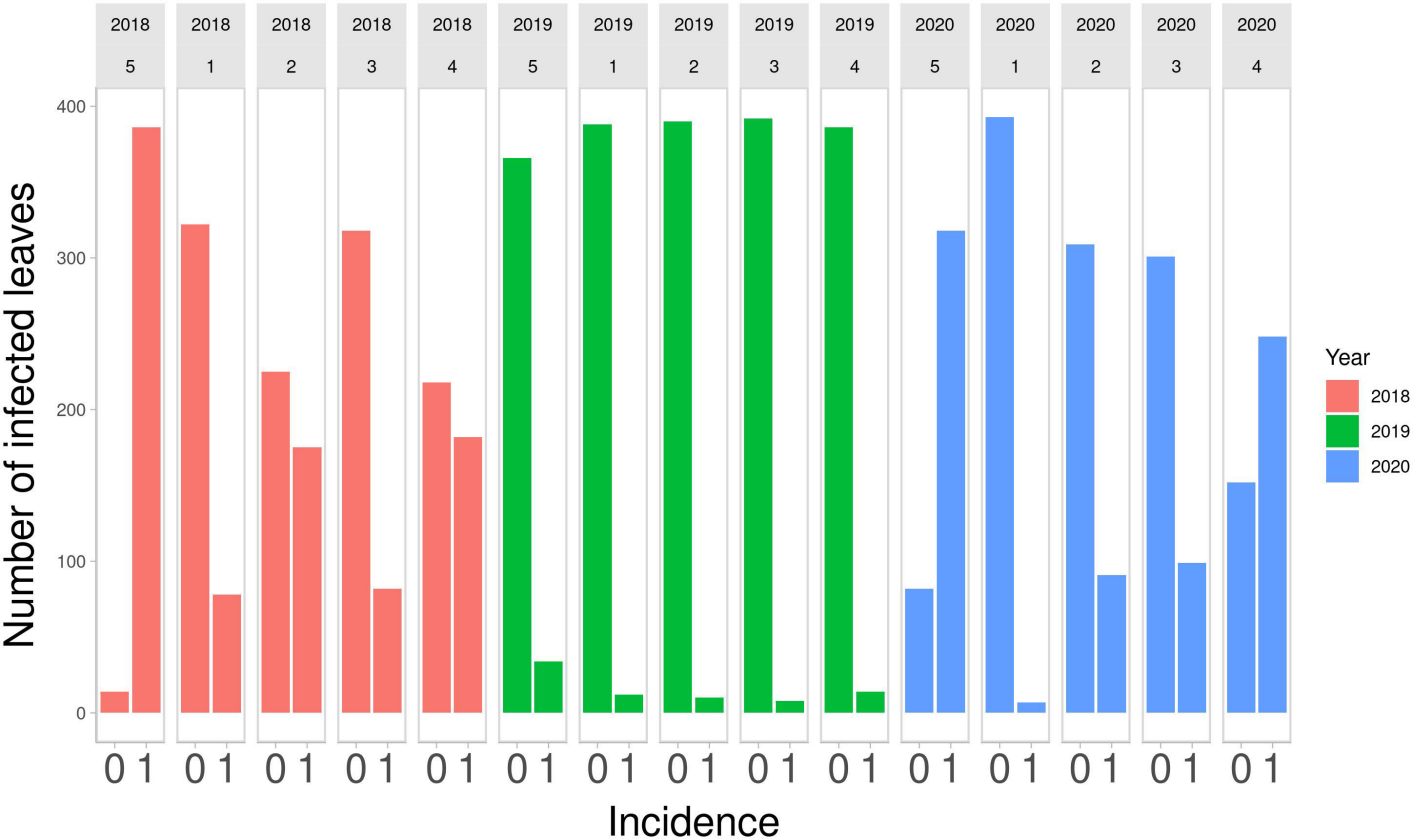
Number of infected leaves out of 400 monitored in the final field survey on Strategy 5 (control), and on other 4 strategies in the 3 years of study. 0, symptom absent; 1, symptom present.

Descriptive statistics were calculated to summarize the distributions of DM over the years of observation. In **Figure 1**, a bar plot of counts of infected and non-infected leaves by strategy is shown, from 2018 to 2020.

The number of infected leaves varies across years. 2018 Strategy 4 and Strategy 2 had a similar number of infected and non-infected leaves, as was the case for Strategy 1 and Strategy 3. Strategy 5 had the highest number of infected leaves. In 2019, there were few infected leaves for all the strategies. Strategy 5 had the highest number of infected leaves. In 2020, Strategy 1 had the lowest number of infected leaves, Strategy 2 and Strategy 3 had a similar number of infected leaves and Strategy 4 had more infected leaves than non-infected leaves. Strategy 5, as expected, had the highest number of infected leaves. The numbers of infected and non-infected leaves are reported quantitatively in **Table 1**. These numbers highlight that Strategy 1 showed the lowest number of infected leaves.

**Table 1:** Absolute frequency of infected (inf) and non-infected (non-inf) leaves observed in each year and strategy.

Year	Status	Strategy 1	Strategy 2	Strategy 3	Strategy 4	Strategy 5
2018	inf.	78	175	82	182	386
2018	non-inf.	322	225	318	218	14
2019	inf.	12	10	8	14	34
2019	non-inf.	388	390	392	386	366
2020	inf.	7	91	99	248	318
2020	non-inf	393	309	301	152	82

### 3.2 A-posteriori distributions and parameter estimates

The *a-posteriori* parameter values are reported in **Table 2**, where betas with indexes from 1 to 4 are reported as odds ratios (OR) and the baseline was Strategy 5, while $\beta_{0}$ represents the odds between the probability of being infected or not for Strategy 5.

**Table 2:** Summary of marginal a-posteriori distributions for each model parameter are shown: mean, quantile 0.025, quantile 0.975, median and highest Maximum A Posteriori (MAP) density estimate.

Parameter	Mean	2.5%	50%	97.5%	MAP
*β*_0_	3.11	0.091	3.11	123.36	3.16
*β*_1_	0.0237	0.0003	0.025	1.76	0.022
*β*_2_	0.080	0.003	0.082	2.32	0.082
*β*_3_	0.052	0.002	0.055	1.45	0.053
*β*_4_	0.170	0.005	0.17	5.27	0.166
*σ_α_*	2.25	0.80	1.96	5.37	1.55
$\sigma_{\gamma_{1}}$	2.32	0.68	1.97	6.01	1.48
$\sigma_{\gamma_{2}}$	1.06	0.05	0.78	3.72	0.58
$\sigma_{\gamma_{3}}$	1.02	0.03	0.74	3.67	0.44
$\sigma_{\gamma_{4}}$	1.32	0.1	1.03	4.12	0.73
$\sigma_{\gamma_{5}}$	2.08	0.6	1.76	5.43	1.31

The parameter $\sigma_{\gamma }$ is the standard deviation of the random parameter that describes a group effect that evaluates how the strategy effect changes every year, while the parameter $\sigma_{\alpha }$ is the standard deviation of the random parameter that describes a group effect that evaluates the year effect changes in the study. For each parameter the mean, quantile at q=0.025 , quantile at q=0.975 , the median and the highest Maximum A Posteriori (MAP) probability estimate are reported.

**Figure 2:**
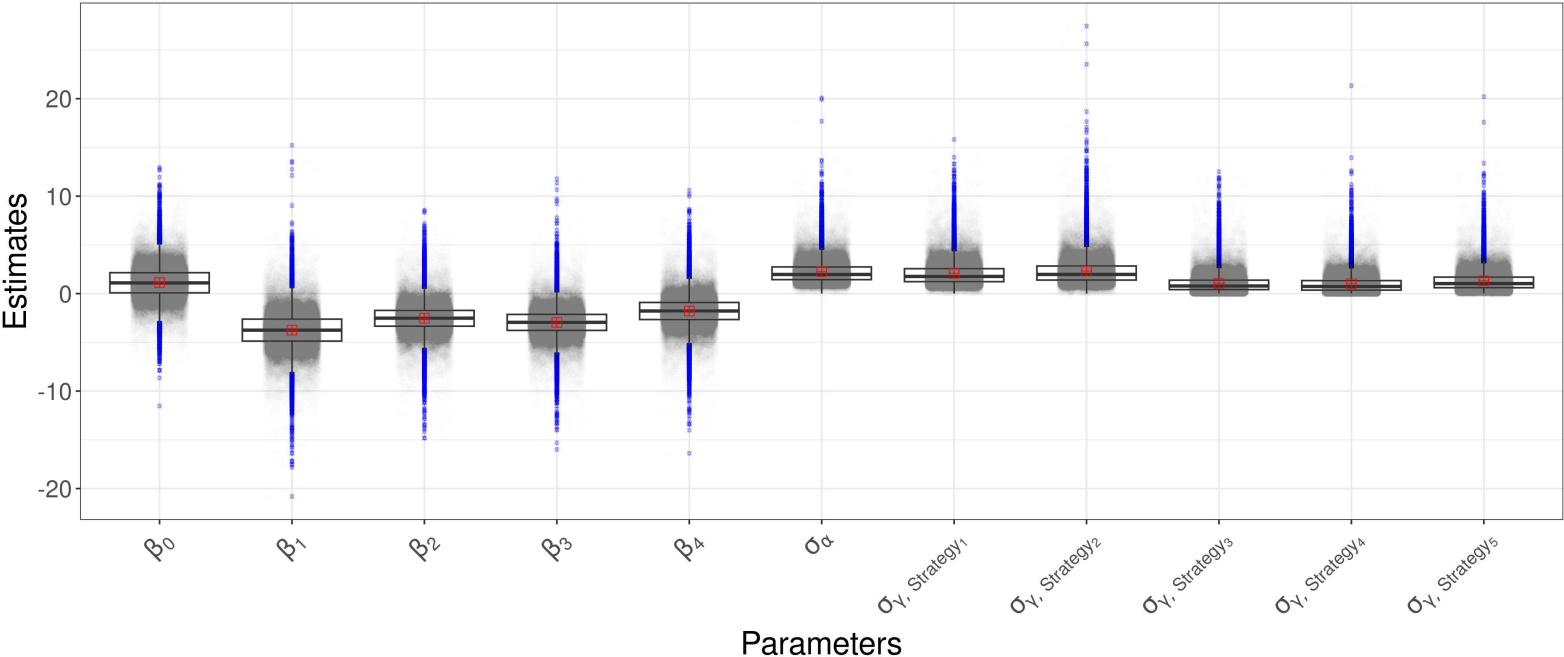
Boxplot of the a-posteriori marginal distributions of model parameters.

The comparison between the OR of $\beta_{1}$ , which represents Strategy 1, against the OR of $\beta_{2}$ , which represents Strategy 2, gives 0.30, indicating the decreased occurrence of disease presence using Strategy 1. The comparison between the OR of $\beta_{3}$ , which represents Strategy 3, against the OR of $\beta_{4}$ , which represents Strategy 4, gives 0.31, indicating the decreased occurrence of disease presence using Strategy 3. The comparison between the OR of $\beta_{1}$ against the OR of $\beta_{3}$ , gives 0.46, indicating the decreased occurrence of disease presence using Strategy 1. The comparison between the OR of $\beta_{1}$ against the OR of $\beta_{4}$ , gives 0.14, indicating the decreased occurrence of disease presence using Strategy 1. The comparison between the OR of $\beta_{2}$ against the OR of $\beta_{3}$ , gives 1.54, indicating the increased occurrence of disease presence using Strategy 2. The comparison between the OR of $\beta_{2}$ against the OR of $\beta_{4}$ , gives 0.47, indicating the decreased occurrence of disease presence using Strategy 2. The 95% intervals range from 0.0003 to 1.76 for $\beta_{1}$ , from 0.003 to 2.32 for $\beta_{2}$ , from 0.002 to 1.45 for $\beta_{3}$ and from 0.005 to 5.27 for $\beta_{4}$ . In **Figure 2** the boxplot of the *a-posteriori* distributions of log_*odds*_ of each parameter are reported. The density distributions were symmetric—indeed, the median and mean had similar values. The standard deviation of α , reported as $\sigma_{\alpha_{t}}$ , had an expected value of 2.25 and its interval ranges from 0.80 to 5.37 and represents the heterogeneity of the year effect. The heterogeneity of each strategy effect is expressed through the parameters $\sigma_{\gamma_{k}}$ , which are reported in **Table 2**. Strategy 1 had the highest heterogeneity—the expected value of its standard deviation was 2.32, followed by Strategy 5 with an the expected value of 2.08, Strategy 4 with an expected value of 1.32, Strategy 2 with an expected value of 1.06 and then Strategy 3 with an expected value of 1.02. The density distributions of standard deviations are reported in **Figure 2**, where it is possible to see that the values are positively skewed. MAP for strategy effect was similar to their expected values, while the MAP of group effect variances was smaller than their expected values, except for the group effect variance of Strategy 5 (control). Summary statistics about group effects and their density distributions are reported as **Supplementary Material**.

$\beta_{1}$ to $\beta_{4}$ represent the fixed effects of the disease management strategies (the Strategy 1-4), $\beta_{0}$ is the baseline and corresponds to the Strategy5, $\sigma_{\alpha }$ is the standard deviation of random effect α describing the random fluctuation due to year, and $\sigma_{\gamma }$ is the standard deviation of random effect γ describing year-specific fluctuations of strategies around the average.

### 3.3 Forecasting of future infection

**Figure 3:**
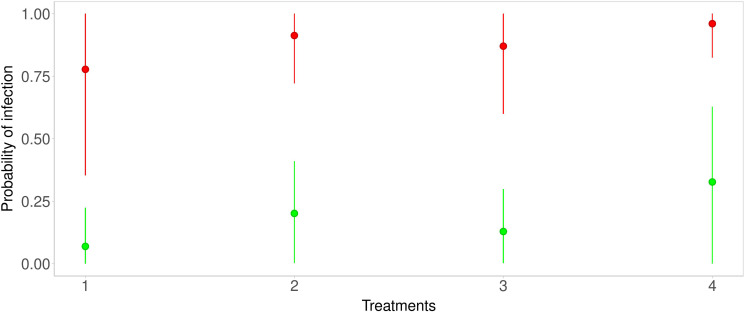
Predictive distributions for each strategy—green points and lines are related to the Average scenario, while red points and lines are related to the Severe scenario.

The predictions were obtained for each considered strategy. Below, figures of the estimated predicted probability density functions are shown together with the Highest posterior Density Interval (HDI) at 80%. In **Figure 3**, the median and the HDI of the predictions for all strategies, and infection probabilities for the Average scenario (green line) and the Severe scenario (red line), are reported. Under the conditions of Strategy 1, infection probability for the Average scenario (green line) ranges from about 0 to about 0.24 (HDI at 80%) with a mean of 0.15. The Severe scenario (red line) infection probability ranges from about 0.35 to about 1 (HDI at 80%) with a mean of 0.68. With Strategy 2, in the Average scenario (green line), infection probability ranges from about 0.002 to about 0.41 (HDI at 80%) with a mean of 0.26. Infection probability for the Severe scenario (red line) ranges from about 0.72 to about 1 (HDI at 80%) with a mean of 0.84. With Strategy 3, in the Average scenario (green line) infection probability ranges from about 0.003 to about 0.3 (HDI at 80%) with a mean of 0.20. The Severe scenario (red line) infection probability ranges from about 0.6 to about 1 (HDI at 80%) with a mean of 0.8. With Strategy 4, in the Average scenario (green line) infection probability ranges from about 0.0007 to about 0.63 (HDI at 80%) with a mean of 0.40. The Severe scenario (red line) infection probability ranges from about 0.82 to about 1 (HDI at 80%) with a mean of 0.90.

### 3.4 Extended evaluation of the Crop protection strategies

The goal of the utility function *U*(*k*) was to identify the crop protection strategy k^*^ against DM infection that achieved the maximum of the expected value with respect to a multi-attribute description of consequences due to the strategy. In **Tables 3** and **4** the expected values of *U*(*k*) for each considered strategy *k* and scenario are reported.

**Table 3:** Utility of the crop protection strategies.

Scenario	Strategy 1	Strategy 2	Strategy 3	Strategy 4	Strategy 5
Average year	0.576	0.250	0.380	0.138	0.031
Severe year	0.038	0.006	0.005	0.004	0.003

Integrated Pest Management (IPM) (“Strategy 1”), the IPM management modified by reduction in fungicides and use of plant defense supporting biostimulants (IPM-GG) (“Strategy 2”), organic management (ORG) (“Strategy 3”), organic management with reduced copper application, and plant defense supporting biostimulants (ORG-GG) (“Strategy 4”) and only biostimulants application (“Strategy 5”).

**Table 4:** Utility of the crop protection strategies after considering the environmental indexes.

Scenario	Strategy 1	Strategy 2	Strategy 3	Strategy 4	Strategy 5
Average year	0.461	0.311	0.455	0.281	0.308
Severe year	0.117	0.125	0.189	0.218	0.236

Integrated Pest Management (IPM) (“Strategy 1”), the IPM management modified by reduction in fungicides and use of plant defense supporting biostimulants (IPM-GG) (“Strategy 2”), organic management (ORG) (“Strategy 3”), organic management with reduced copper application, and plant defence supporting biostimulants (ORG-GG) (“Strategy 4”) and only biostimulants application (“Strategy 5”).

Results suggest that Strategy 1 was the most effective against DM infection for both scenarios. In the Average scenario, Strategy 3 was more effective than Strategy 2, and the least effective strategy against DM infection was Strategy 4. In the Severe scenario, Strategy 2 was more effective than Strategy 3, and the least effective strategy against DM infection was Strategy 4.

Strategy 1 was still the most effective in the Average scenario, followed by Strategy 3. In the Severe scenario, among those considered here, the biostimulants strategy was the most effective in terms of expected utility. It is important to emphasize that the optimal decision depends heavily on the expert-specific definition of the utility function. Indeed, by changing its parameters different results can be achieved. In this case it seems that after reaching a specified threshold, the best decision to take is simply to support plant vigor.

But the results change fully after the introduction of the environmental component utility values, which showed that the strategies were closer to each other. Indeed, utilities of Strategy 1 and Strategy 3 were 0.461 vs 0.455 instead of 0.576 vs 0.380 and utilities of Strategy 2 and Strategy 4 were 0.311 vs 0.281 instead of 0.380 vs 0.138. It would seem that the environmental components gave a boost in terms of utility to strategies that had a lower environmental impact. Indeed Strategy 1 utility decreased (0.576 to 0.461) and Strategy 3 utility increased (0.380 to 0.455).

### 3.5 Model diagnostics

**Figure 4:**
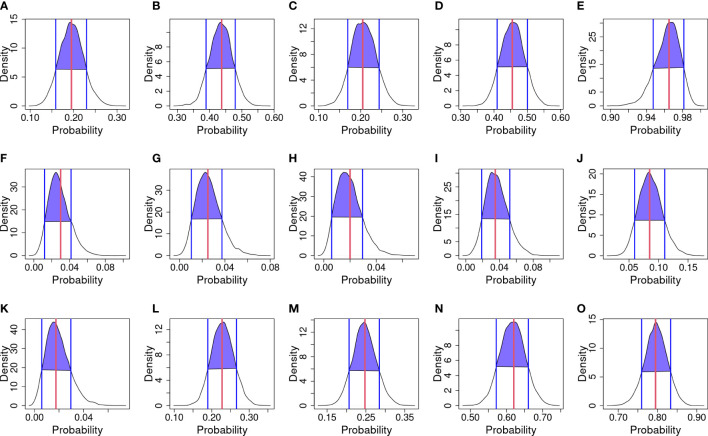
Posterior predictive checks. The black curve line represents the kernel density of predicted probabilities of infection for each year and strategy; blue vertical lines and purple area are the Highest density interval (HDI) at 80%; and the red vertical line is the infection probability observed. **(A)** 2018 & Strategy 1. **(B)** 2018 & Strategy 2. **(C)** 2018 & Strategy 3. **(D)** 2018 & Strategy 4. **(E)** 2018 & Strategy 5. **(F)** 2019 & Strategy 1. **(G)** 2019 & Strategy 2. **(H)** 2019 & Strategy 3. **(I)** 2019 & Strategy 4. **(J)** 2019 & Strategy 5. **(K)** 2020 & Strategy 1. **(L)** 2020 & Strategy 2. **(M)** 2020 & Strategy 3. **(N)** 2020 & Strategy 4. **(O)** 2020 & Strategy 5.

Graphical diagnostics were calculated in order to assess model performances—posterior predictive probability and their HDI are shown in **Figure 4**. The curves represent the probability of infection drawn from the model; the red line in each panel represents the observed mean of infection; while blue lines and blue areas represent the HDI.

Considering 2018 (**Figures 4A–E**) observed mean matches the mean of draws, except for Strategy 3 where a bimodal trend in kernel density is observed. In 2019 (**Figures 4F–J**), kernel densities are shifted to the left to respect the observed mean. In 2020 (**Figures 4K–O**), the observed mean matches the mean of draws. Considering the HDI for Strategy 1, in 2018 (**Figure 4A**) the interval ranges from about 0.16 to 0.24; in 2019 (**Figure 4F**) the interval ranges from about 0.015 to 0.04; and in 2020 (**Figure 4K**) the interval ranges from about 0.01 to 0.025. Considering the HDI for Strategy 2, in 2018 (**Figure 4B**) the interval ranges from about 0.38 to 0.48; in 2019 (**Figure 4G**) the interval ranges from about 0.015 to 0.04; and in 2020 (**Figure 4L**) the interval ranges from about 0.18 to 0.26. Considering the HDI for Strategy 3, in 2018 (**Figure 4C**) the interval ranges from about 0.16 to 0.24; in 2019 (**Figure 4H**) the interval ranges from about 0.01 to 0.03; and in 2020 (**Figure 4M**) the interval ranges from about 0.20 to 0.28. Considering the HDI for Strategy 4, in 2018 (**Figure 4D**) the interval ranges from about 0.41 to 0.50; in 2019 (**Figure 4I**) the interval ranges from about 0.02 to 0.05; and in 2020 (**Figure 4N**) the interval ranges from about 0.57 to 0.60. Considering the HDI for Strategy 5 in 2018 (**Figure 4E**), the interval ranges from about 0.95 to 0.98; in 2019 (**Figure 4J**) the interval ranges from about 0.06 to 0.11; and in 2020 (**Figure 4O**) the interval ranges from about 0.75 to 0.84.

**Figure 5:**
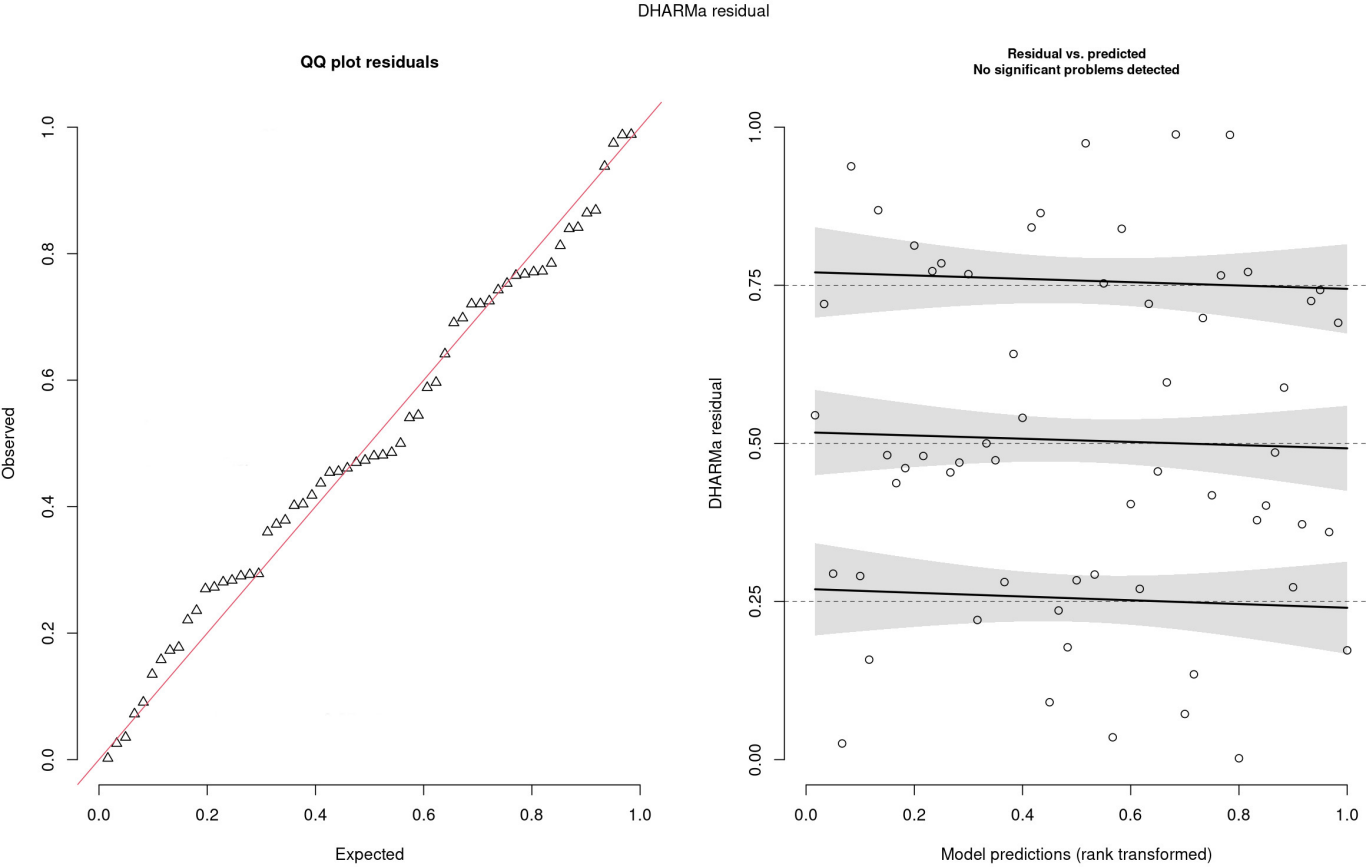
Analysis of DHARMa residuals.

**Figure 6:**
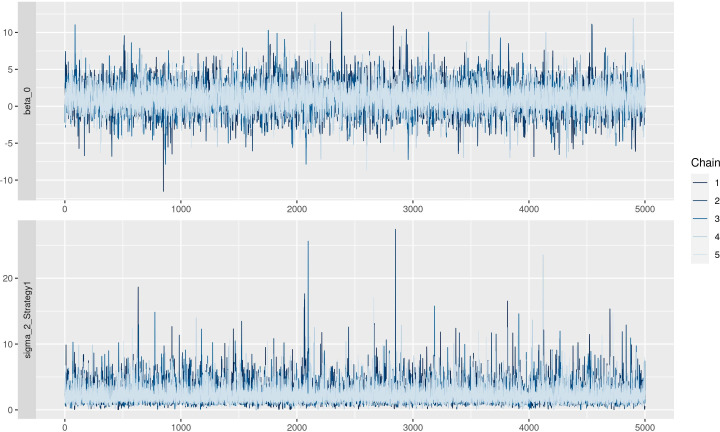
Traceplot of Markov Chain Monte Carlo simulations.

The residuals are reported in **Figure 5**. In the left panel, the QQ plot of the residual was reported, and no problems were highlighted since residuals follow the red line, meaning that there was no relevant difference between observed and expected values. In the right panel, standardized residuals were plotted vs model predictions, but no trend was observed since regression lines (black line) were almost parallel. Traceplots of the HMC sampler are reported in **Figure 6**. All traceplots showed the same behavior, so there is little reason to call into question the performance of the algorithm. Therefore only two traceplots are reported here.

## 4 Discussion

In our analysis, we applied a Bayesian model developed for the vineyard under study, or any other vineyard with similar environmental characteristics. The model is suited to comparing different protection protocol strategies against *Plasmopara viticola*, and to predicting the probability of infection after strategy, providing key information for selecting the best crop strategy among the following: IPM, IPM-GG, ORG, ORG-GG or application of biostimulant products alone. The latter was used as the control strategy of this study, not only because a full negative control (no intervention of any type) was absent due to the large size of each plot, but also because the application of biostimulants is intended to stimulate plant immune systems to become more effective against pathogens, and therefore by definition they do not have a direct effect on pathogen growth itself [(Shahrajabian et al., 2021, Bertrand et al., 2021 and La Spada et al., 2021)]

The Bayesian model was applied while taking into account the main sources of heterogeneity of the phenomenon. Indeed, in the model specification, two group-level effects were considered. As described in Model specification, α_t_ was specified to take into account the different disease pressure on plants each year. This can be observed also in **Figure 1** and **Table 1** in Strategy 5 bars and columns—indeed, in 2018 the proportion of infected leaves was 96.5%, in 2019 this figure was 8.5%, and in 2020 it was 79.5%. Considering that sporangia are a typical component of the airborne microflora, these differences in the infection percentage could be due to meteorological conditions (Brischetto et al., 2020). Indeed, weather data from Perria et al. (2022) showed that 2018 was particularly positive for DM development—more than in 2019—due to the fewer leaf wetness hours, which is very important for DM development. Since the model did not include data from meteorological conditions, the estimation of *α_t_* and its standard deviation (*σ_α_* = 2.25) could provide for the variability of disease pressure due to favorable or unfavorable meteorological conditions, acting as a proxy variable which describes the disease pressure. For the same reason, we specified a parameter *γ_k,t_* to take into account the variability of the protocols affect every year. Considering the assignment of strategies to plots, we were constrained by the experimental design originally defined for an already performed experiment. Our reanalysis is in any case suited to the quite large area considered because local experts clearly stated that this specific vineyard is reasonably homogeneous, with the same type of soil, the same slope, and the same exposure. We obviously agree that randomization is to be preferred in general, but we maintain that is not crucial point here. No data about microclimate or soil analyses were collected, thus we considered a model with subplots as random effects in order to estimate the standard deviation. In the analysis, such a model was considered, but estimated standard deviations of random effects for each strategy in each subplot were quite low (see **Table S2** for results). The leave-one-out (LOO) cross-validation (Vehtari et al., 2017), was used to compare the considered models, and it confirmed that introducing subplots did not improve the predictive performance of the model, which is why subplots have been removed from the final model. Therefore, following the LOO, as well as the degree of belief of our expert, we peacefully stated that our vineyard is quite homogeneous. In any case, our statement should not be interpreted as a (bad) suggestion of avoiding randomization or even neglecting heterogeneity at all in general. The comparisons in terms of LOO between the model with and without the subplots as predictors are reported in **Table S3**. As reported in Forecasting of future infection, Strategy 1, which corresponds to IPM, gave the best prediction in both scenarios. Indeed, its predictive probability mass was concentrated around 15% in the Average scenario and 68% in the Severe scenario. **Figure 3** highlights that probability distributions had a high dispersion, especially in the Severe scenario, in which high uncertainty of prediction is inherent. This could be due to the behavior of the disease in the 3-year study. Indeed as reported in Descriptive statistics, in 2019 a very low amount of disease was observed (8.5%). Moreover, high dispersion could be due to the absence of meteorological variables in the model specification. This could be confirmed by Chen et al. (2020) who used GLM (with a frequentist approach) to predict DM on leaves. Their results suggest that data about rainfall, especially recorded in March and April, were important to predict occurrence of the disease on leaves. The oospore germination process leading to macrosporangia production, which is the disease inoculum responsible for primary infections, is strongly inhibited where dry springs occur. Despite the relevant importance of the meteorological variable, in the discussion section, the authors recommend the usage of GLM where only the dates of disease onset detected by monitoring were used as an explanatory variable. In the case of Chen et al. (2020), meteorological variables were not available before June but despite this, their absence in the model specification did not compromise model performances. So, even if the disease is a function of meteorological data, the observation of its actual development in the field is enough to overcome the missing climatic information. This conjecture supports our approach based on group-level parameters as proxy variables that quantify differences in disease pressure and therefore explain the variability of the disease pressure due to favorable-unfavorable meteorological conditions discussed above.

Despite the fact that the predictive probability of infection is a key value in selecting the best strategies, nowadays it is more and more important to take a decision after also considering the environmental impact of the strategy and further possible side effects. In the last part of this work, a multi-attribute approach has been proposed, where variables that describe the environmental impact and the potential of causing human diseases jointly contribute to the optimal decision, namely the selection of the best crop protection strategy. It is important to note that the probability of infection for one future leaf has been calculated using a Bayesian predictive distribution conditional on collected data, while the future environmental impact and side effects were accounted for by a prior-predictive distribution (Multinomial-Dirichlet) mostly dependent on accumulated expert knowledge instead of on extensive data. In the prior-predictive approach, the mean of the only two observed scores per year was considered as the future modal value of the score, and indeed the vector α led to the concentration of probability mass on that observed value. The selected classes belong to the year 2020, because it was considered our average year (Average scenario) compared to the others.

The utility function elicited for presence of the disease depends on a parameter, *δ*, that was set to 0.4, but that value can be changed according to how fast the utility is increased by increasing the probability of a healthy leaf, i.e. by expert judgment: if the δ is less than 1 then the resulting value decreases quickly while if the *δ* is greater than 1 the result decreases slowly, as flexibility is required to adapt to expert-specific evaluations and differences in vineyards. In this work, the numerical weights assigned to the various attributes were determined based on their relevance for utility, which can vary depending on the purpose of the study and the preferences of the decision-makers. Indeed, Lavik et al. (2020) applied the SMART approach in an agronomic context and studied many scenarios with a different set of numerical weights, showing that changing weights can strongly change the outcome. In this work, results from expected utility show that the inclusion of the environmental attributes had an impact on the outcome: indeed when they were excluded, the IPM strategy dominated all other strategies in the average scenario. On the other hand, when they were included, utility values between IPM and IPM-GG became closer because of the low environmental impact of the “Green Grapes” version, especially for the Eco Tox score. Since both ORG and ORG-GG increased their utility values after including environmental attributes, the distance in terms of utility was the same, meaning that there were no differences in terms of sustainability, but only in terms of predictive disease detection. The observation of individual attributes highlighted that there were actually some differences. Indeed ORG-GG had a higher utility value for Carbon footprint and Carbon sequestration (data not shown), but a lower value for the Human tox score compared to ORG. However, the overall utility for the environmental components was the same. In the Severe scenario, a biostimulants-only approach (Strategy 5) was the best strategy—a result suggesting that when disease pressure is very high due to favorable climatological conditions then the use of the other strategies is not enough to counter the disease, without a high environmental impact. The latter result is strictly dependent on the elicitation of the sub-utility functions. Indeed, changing the tuning parameters, for instance by increasing the threshold of the sub-utility function describing future infections, might decrease the dominance of Strategy 5 in the Severe scenario. Therefore, since there is no unique-natural utility function, different agronomists can customize the sub-utility functions according to their attitude to risk, their evaluation of environmental side effects, as well as current regulatory dispositions, and thus leading to different optimal decisions. Despite IPM and ORG being the best strategies in the Average scenario, their utilities were not dominant, therefore an agronomist could change weights further to reward the “GreenGrapes” strategy that guarantees greater environmental sustainability of viticulture. These results can contribute greatly to a more targeted approach in disease control management, by selecting products with lower environmental impact based on risk assessment, aligning with current European guidelines for plant protection. Instead of synthetic products that have a high environmental impact, the use of substances that induce plant defense, basic compounds, and plant strengtheners with low environmental impact is recommended. However, the application of these alternatives, especially under a lower disease pressure, benefits considerably from the support of models in interpreting risk and guiding the selection of these less potent yet environmentally friendly products.

In contrast to mechanistic-deterministic approaches recently published in the literature which are based on differential equations, we have proposed a statistical approach grounded in accumulated real-world expertise and probabilistic evaluation of uncertainty. This key feature, besides enabling more flexibility in the analysis, also entails certain limitations. First, the quality of predictions strongly depends on sample size and on the extent of the natural variability in collected data. In a full Bayesian approach, variability is almost never neglected, thus bold overconfident statements are typically not a risk, but at the same time large samples are needed to reduce uncertainty to a practically useful degree. Second, in our work we exploited expert knowledge while defining assumptions for our model, but we did not use highly informative prior distributions for model parameters. Nevertheless, an analysis in which an experienced expert defines highly informative prior distributions remains a possibility for future work, given that in our case we have chosen to let data “speak aloud”. Third, uncertainty in prediction was not always small, a feature that we tend to prefer compared to the alternative of artificial overprecision and risky decisions. Fourth, our model did not consider the mechanistic features of the underlying causal data-generating process. We conjecture that the proposed statistical model could almost surely be improved by combining mechanistic and statistical approaches into a unified framework: deterministic models could play the role of anchors while defining structural causal models task that is likely to require specifically planned studies, as we have stated previously in our recent work Stefanini and Valleggi (2022).

## 5 Conclusion

*Plasmopara viticola* is the causal agent of downy mildew, one of the most damaging diseases of grapevines. A model able to select the best strategy against downy mildew could be a suitable tool in order to choose the optimal strategy based on the local characteristics of the vineyard, in terms of disease pressure and spread. In this work, a Bayesian decisional approach was used in order to combine different sources of information and select the best strategy for the next year of grapevine production, considering at the same time the efficiency of the strategy and its environmental impact. Thanks to the proposed utility function, the agronomist may consider several attributes on a very easily interpretable scale. Furthermore, it is also possible to change the emphasis of the analysis choosing weights to obtain the best balance between environmental attributes and strategy efficiency as a result of risk attitude and interest in sustainability that characterizes the decision maker.

In order to improve this tool, more than three years of study are required due to the presence of high seasonal variability. For example, in 2019 very low numbers of infected leaves were observed due to unfavorable meteorological conditions for the pathogen. The natural next step of this framework would be an extension of the proposed utility function where more attributes are considered, in particular by introducing attributes describing the quality and the disease incidence of grapes, the economic aspect of each strategy, and also considering the joint assessment of utility value over attributes, e.g. considering utility dependence within some subsets of attributes.

## Data availability statement

The original contributions presented in the study are publicly available. This data can be found here: source data can be downloaded from DataVerse at UNIMI https://dataverse.unimi.it/dataverse/unimi/?q=stefanini, dataset and R code: https://github.com/federico-m-stefanini.

## Author contributions

LV: Writing – original draft, Visualization, Software, Methodology, Formal analysis, Conceptualization. GC: Writing – review & editing, Validation, Resources, Investigation. RP, LM: Writing – review & editing, Validation, Supervision, Resources, Investigation. FS: Writing – review & editing, Supervision, Methodology, Conceptualization.
