# Supplementary material for: A Bayesian model for control strategy selection against Plasmopara viticola infections
Source: Front Plant Sci. 2023 Jul 20;14:1117498. doi: 10.3389/fpls.2023.1117498 (PMC10399454; doi:10.3389/fpls.2023.1117498)
Supplement: Supplementary file 1 [file DataSheet_1.pdf]

Supplementary Material 1

1 Supplementary Figures and Tables

1.1 Supplementary Figures

Supplementary Material

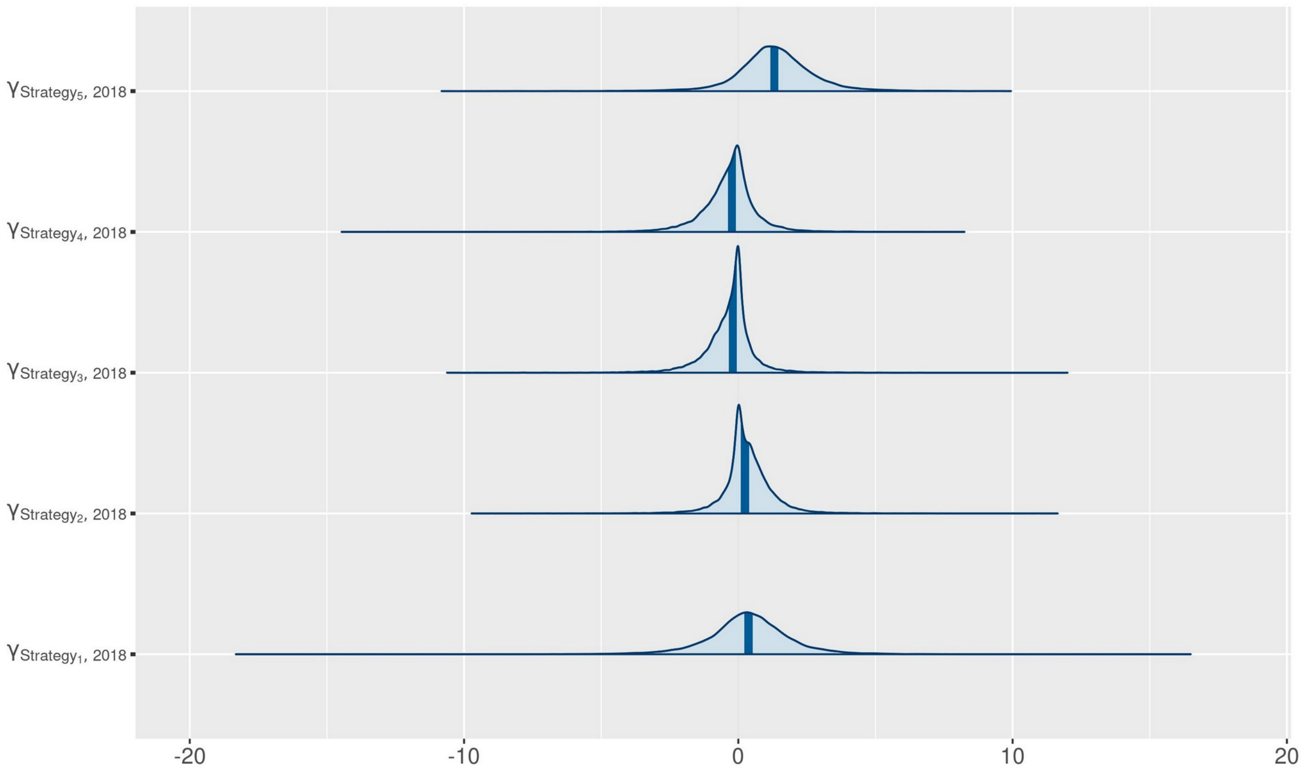

Figure S1 A-posteriori distributions of the random effect describing year-specific fluctuations of strategies around the average, in this case year 2018

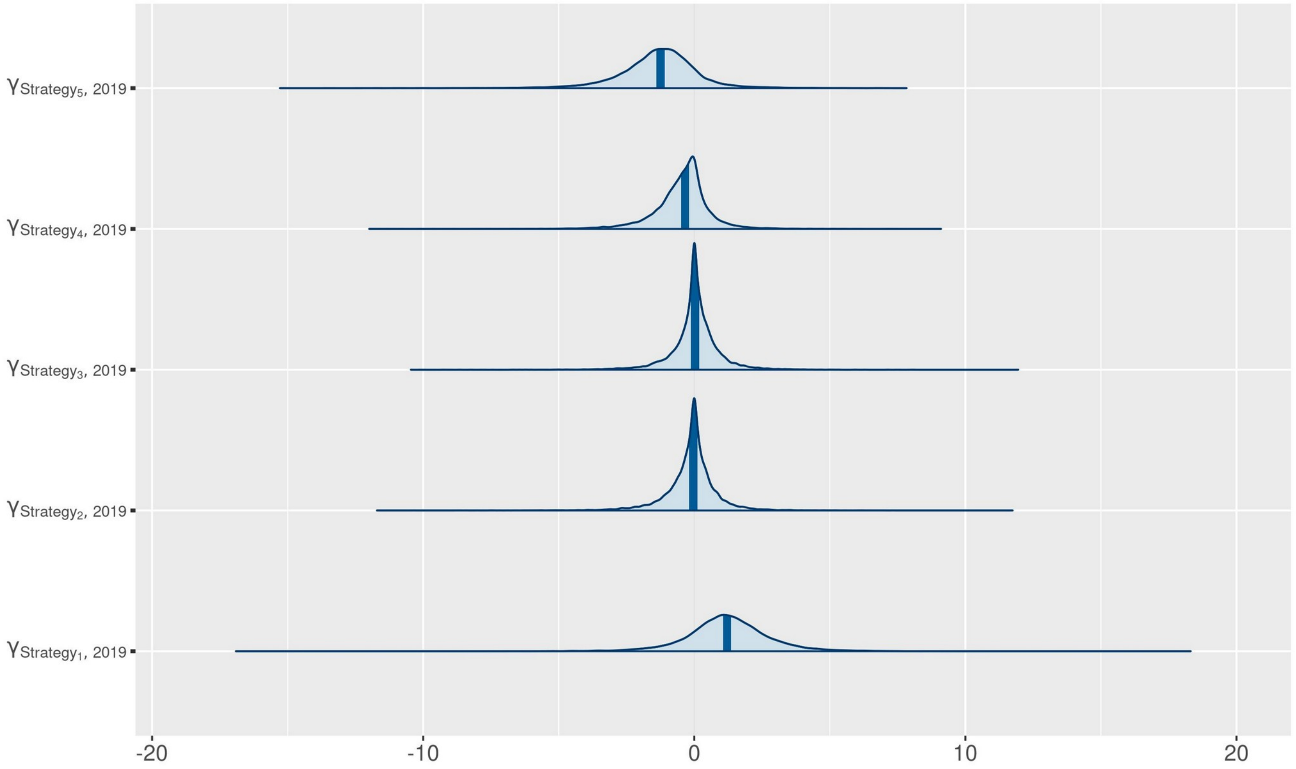

Figure S2:A-posteriori distributions of the random effect describing year-specific fluctuations of strategies around the average, in this case year 2019

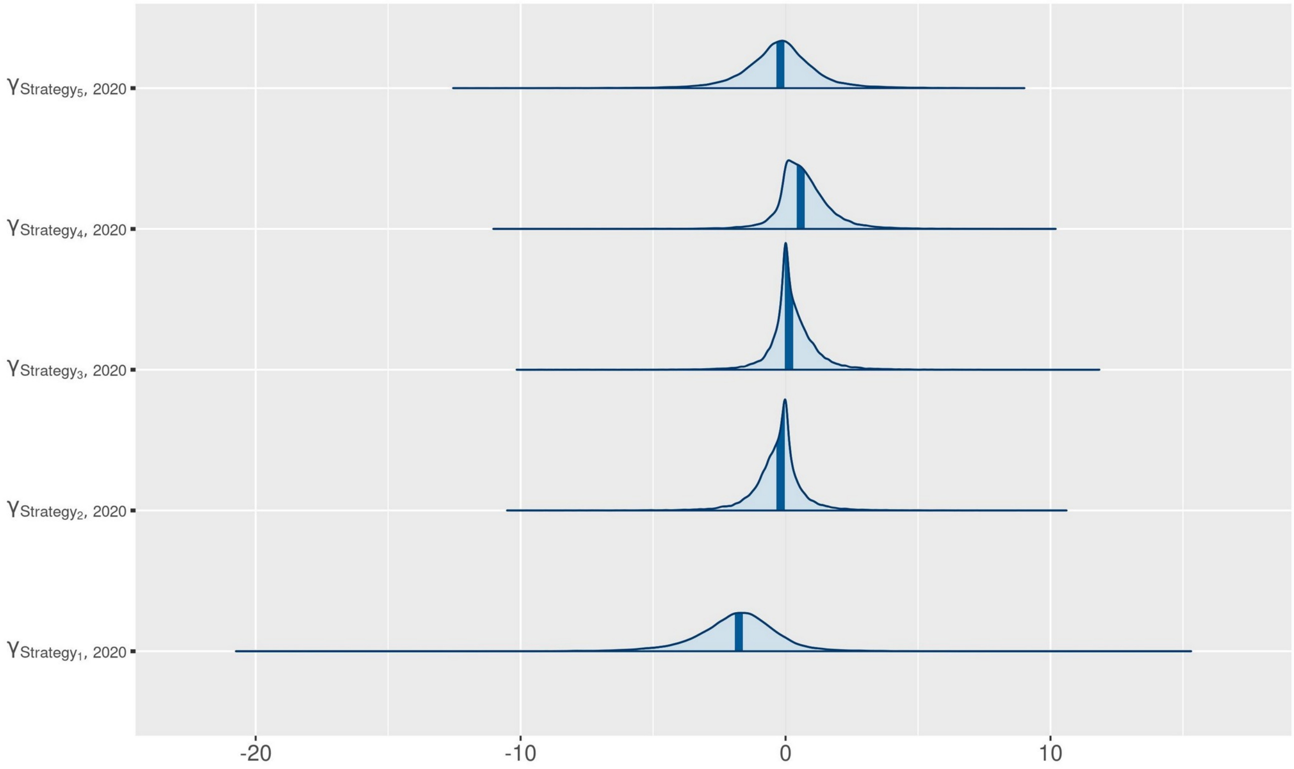

Figure S3:A-posteriori distributions of the random effect describing year-specific fluctuations of strategies around the average, in this case year 2020

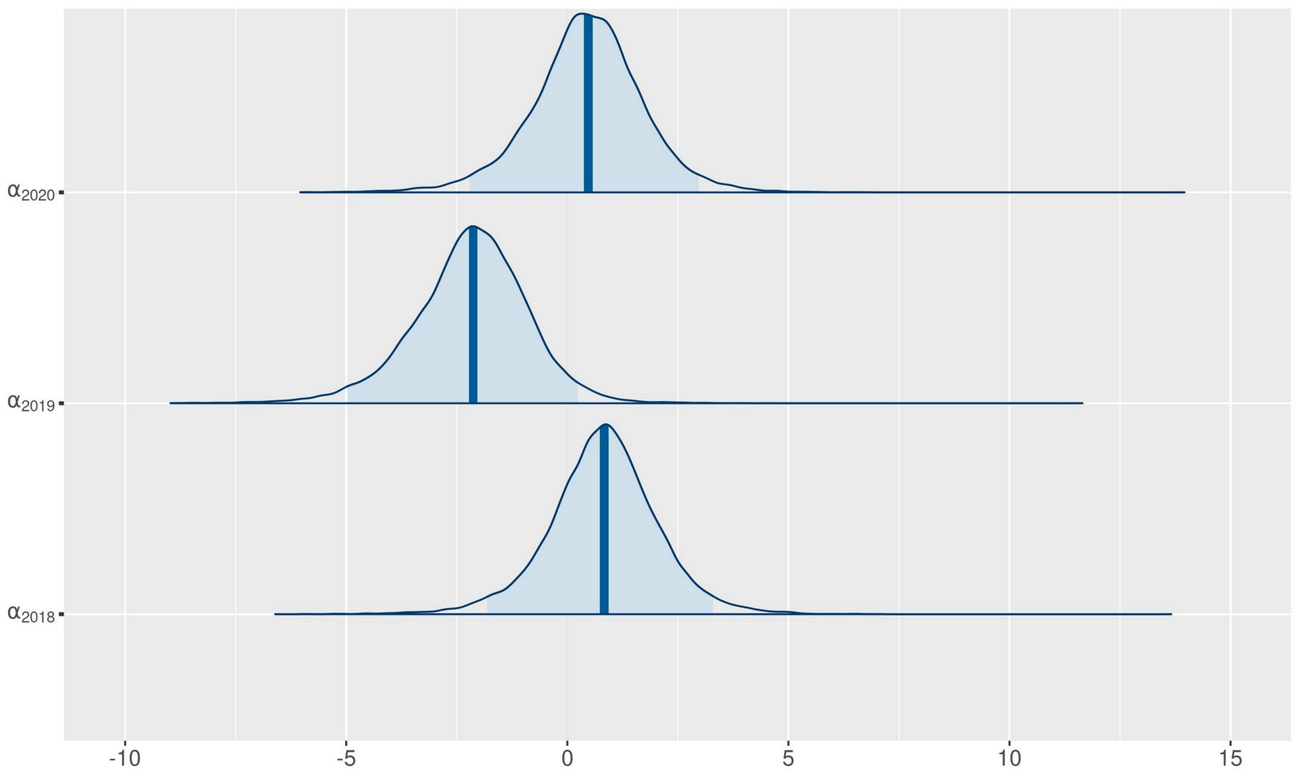

Figure S4:A-posteriori distributions of the random effect the random fluctuation due to year.

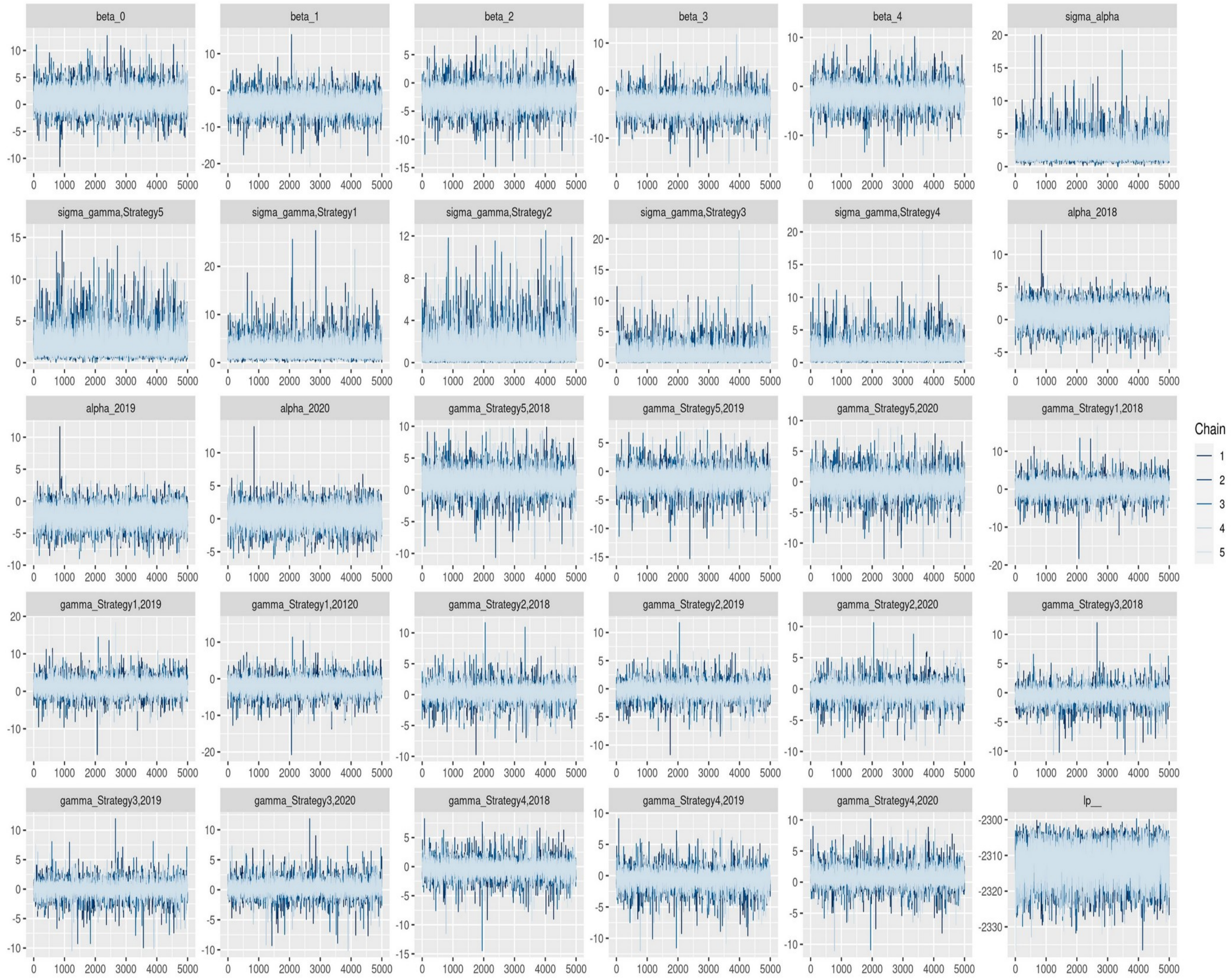

Figure S5: Traceplot of Markov Chain Monte Carlo simulations.

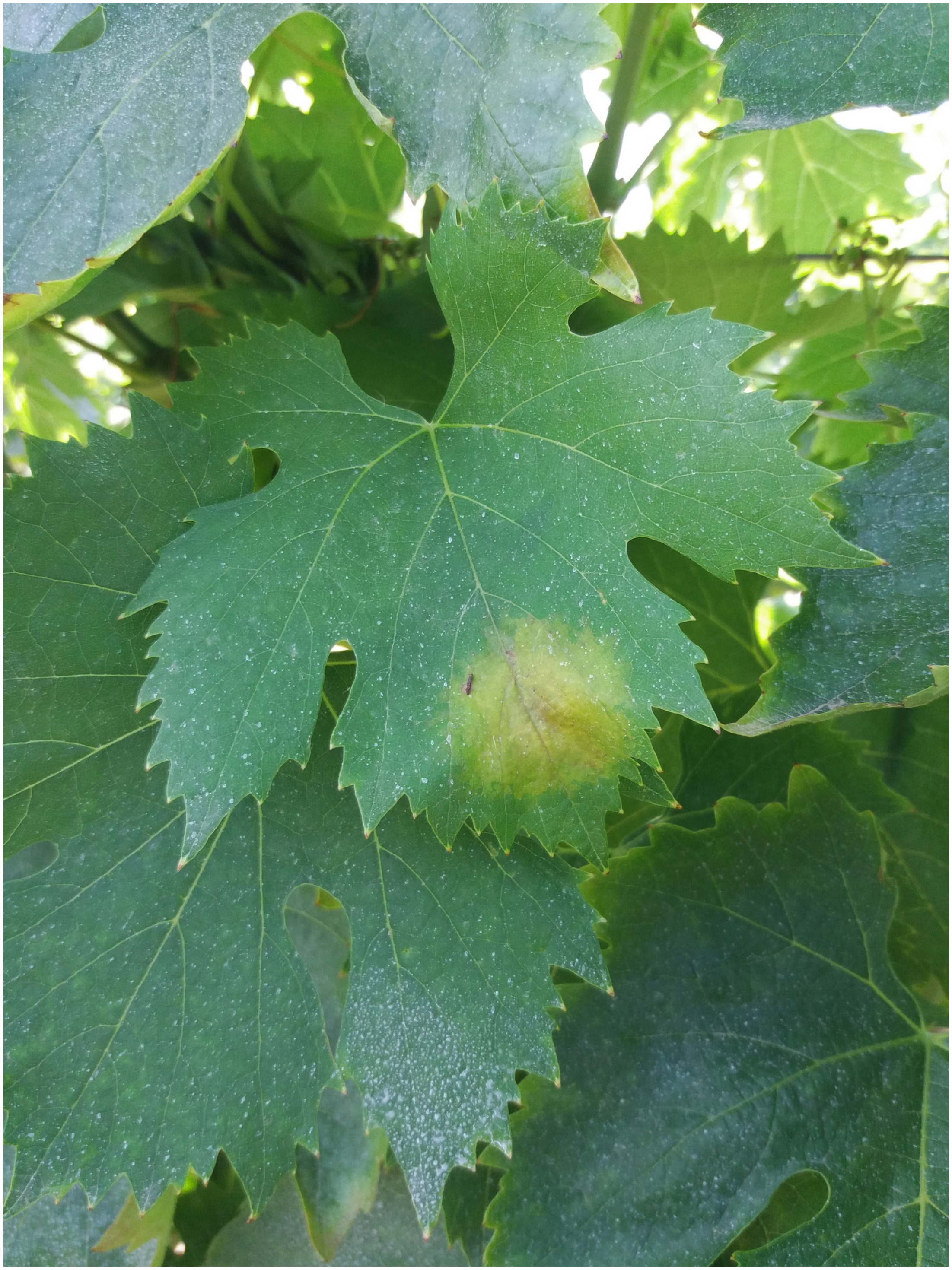

Figure S6: Synthomps of Plasmopara viticola

Table S1: A-posteriori parameter values of the mean, Median, credible intervals and MAP (Maximum A Posteriori)

Parameter	Mean	2.5%	50%	97.5%	MAP
α_{2018}	0.8	-1.8	0.8	3.3	0.86
α_{2019}	-2.2	-5	-2.1	0.2	-2.12
α_{2020}	0.5	-2.2	0.5	3	0.33
$\gamma_{strategy\ 1,2018}$	0.4	-2.9	0.4	3.6	0.33
$\gamma_{strategy\ 2,2018}$	0.3	-1.5	0.2	2.3	0.01
$\gamma_{strategy\ 3,2018}$	-0.3	-2.4	-0.2	1.3	-0.00481
$\gamma_{strategy\ 4,2018}$	-0.3	-2.6	-0.2	1.7	-0.01
$\gamma_{strategy\ 5,2018}$	1.4	0.6	1.3	4.5	1.15
$\gamma_{strategy\ 1,2019}$	1.2	-2	1.2	4.5	1.10
$\gamma_{strategy\ 2,2019}$	-0.1	-2.3	0	1.7	-0.00737
$\gamma_{strategy\ 3,2019}$	0	-2	0	1.9	-0.00716
$\gamma_{strategy\ 4,2019}$	-0.5	-2.9	-0.3	1.5	-0.01
$\gamma_{strategy\ 5,2019}$	-1.3	-4.5	-1.2	1.5	-1.21
$\gamma_{strategy\ 1,2020}$	-1.8	-5.4	-1.8	1.2	-1.68
$\gamma_{strategy\ 2,2020}$	-0.3	-2.4	-0.2	1.6	-0.0045
$\gamma_{strategy\ 3,2020}$	0.2	-1.5	0.1	2.3	-0.0006
$\gamma_{strategy\ 4,2020}$	0.7	-1.2	0.6	3.1	0.03
$\gamma_{strategy\ 5,2020}$	-0.2	-3.3	-0.2	2.8	-0.13

Table S2: A-posteriori parameter values of the mean, standard deviation, median,and credible intervals of the standard deviation of Subplots incidence in each Strategy.

Parameter	Mean	SD	2.5%	50%	97.5%
$sd_{Subplot, Strategy 1}$	0.2	0.2	0.0	0.1	0.7
$sd_{Subplot, Strategy 2}$	0.3	0.4	0.0	0.2	1.2
$sd_{Subplot, Strategy 3}$	0.3	0.3	0.0	0.2	1.2
$sd_{Subplot, Strategy 4}$	0.4	0.4	0.0	0.3	1.5
$sd_{Subplot, Strategy 5}$	0.3	0.4	0.0	0.2	1.3

Leav-one-out cross validation consists in removing data point from the dataset and then estimate the model and predictions, producing an expected log pointwise predictive density (elpd), which is the integration between likelihood and the new posterior computed without a data point, usually in log scale. The model with higher expected log pointwise predictive density is the best one, in this case, the model with subplots had a lower value of this estimate than the model without subplots (the model that we described in the manuscript). Other quantities were estimated that support these results, such as the value of the elpd minus the number of effective parameters in the model (looic). The model with the lower value is the best one, in this case, the model without subplots in the specification had a lower value of this estimate than the model with subplots.

Table S3:Leave-one-out (LOO) cross-validation between models which different specifications, Model 1 has no subplot as predictor for the incidence of leaves. While Model 2 has subplot as predictor for the incidence of leaves. Here are reported the expected log pointwise predictive density (elpd), the effective number of parameters (p_loo), and the value of the elpd minus the number of effective parameters in the model (looic).

Model	elpd	p_loo	looic
$Model 1$	-2284	15.04	4569
$Model 2$	-2286	24.30	4573

List of R packages

- stats
- graphics
- grDevices
- utils
- datasets methods   base
- bayestestR_0.11.5
- posterior_1.1.0
- latex2exp_0.9.4
- arm_1.12-2
- lme4_1.1-27.1
- Matrix_1.4-0
- MASS_7.3-55
- loo_2.4.1
- modelr_0.1.8
- bayesplot_1.8.1
- ROCR_1.0-11
- gganimate_1.0.7
- cowplot_1.1.1
- tidybayes_3.0.1
- lubridate_1.8.0
- brms_2.16.3
- Rcpp_1.0.8
- forcats_0.5.1

- stringr_1.4.0
- dplyr_1.0.7
- purrr_0.3.4
- readr_2.1.2
- tidyr_1.1.4
- tibble_3.1.6
- tidyverse_1.3.1
- rstan_2.21.3
- ggplot2_3.3.5
- StanHeaders_2.21.0-7
- readxl_1.3.1
- backports_1.4.1
- plyr_1.8.6
- igraph_1.2.10
- splines_4.1.3
- svUnit_1.0.6
- crosstalk_1.2.0
- rstantools_2.1.1
- inline_0.3.19
- digest_0.6.29
- htmltools_0.5.2
- rsconnect_0.8.25
- fansi_1.0.2
- magrittr_2.0.2
- checkmate_2.0.0

- tzdb_0.2.0
- RcppParallel_5.1.4
- matrixStats_0.61.0
- xts_0.12.1
- prettyunits_1.1.1
- colorspace_2.0-2
- rvest_1.0.2
- ggdist_3.0.1
- haven_2.4.3
- xfun_0.30
- callr_3.7.0
- crayon_1.5.0
- jsonlite_1.8.0
- zoo_1.8-9
- glue_1.6.2
- gtable_0.3.0
- distributional_0.2.2
- pkgbuild_1.3.1
- abind_1.4-5
- scales_1.1.1
- mvtnorm_1.1-3
- DBI_1.1.2
- miniUI_0.1.1.1
- xtable_1.8-4
- progress_1.2.2

- diffobj_0.3.5
- stats4_4.1.3
- DT_0.20
- datawizard_0.3.0
- htmlwidgets_1.5.4
- httr_1.4.2
- threejs_0.3.3
- arrayhelpers_1.1-0
- ellipsis_0.3.2
- pkgconfig_2.0.3
- farver_2.1.0
- dbplyr_2.1.1
- utf8_1.2.2
- labeling_0.4.2
- tidyselect_1.1.2
- rlang_1.0.2
- reshape2_1.4.4
- later_1.3.0
- munsell_0.5.0
- cellranger_1.1.0
- tools_4.1.3
- cli_3.2.0
- generics_0.1.1
- gifski_1.4.3-1
- broom_0.7.10

- ggridges_0.5.3
- evaluate_0.15
- fastmap_1.1.0
- yaml_2.3.5
- rticles_0.23
- processx_3.5.2
- knitr_1.37
- fs_1.5.2
- nlme_3.1-155
- mime_0.12
- xml2_1.3.3
- compiler_4.1.3
- shinythemes_1.2.0
- rstudioapi_0.13
- reprex_2.0.1
- tweenr_1.0.2
- stringi_1.7.6
- ps_1.6.0
- Brobdingnag_1.2-6
- lattice_0.20-45
- nloptr_1.2.2.3
- markdown_1.1
- shinyjs_2.0.0
- tensorA_0.36.2
- vctrs_0.3.8

- pillar_1.7.0
- lifecycle_1.0.1
- bridgesampling_1.1-2
- insight_0.16.0
- httpuv_1.6.4
- R6_2.5.1
- promises_1.2.0.1
- gridExtra_2.3
- codetools_0.2-18
- boot_1.3-28
- colourpicker_1.1.1
- gtools_3.9.2
- assertthat_0.2.1
- withr_2.5.0
- shinystan_2.5.0
- parallel_4.1.3
- hms_1.1.1
- grid_4.1.3
- coda_0.19-4
- minqa_1.2.4
- rmarkdown_2.13
- shiny_1.7.1
- base64enc_0.1-3
- dygraphs_1.1.1.6
